# Recent Advances in Donepezil Delivery Systems via the Nose-to-Brain Pathway

**DOI:** 10.3390/pharmaceutics17080958

**Published:** 2025-07-24

**Authors:** Jiyoon Jon, Jieun Jeong, Joohee Jung, Hyosun Cho, Kyoung Song, Eun-Sook Kim, Sang Hyup Lee, Eunyoung Han, Woo-Hyun Chung, Aree Moon, Kyu-Tae Kang, Min-Soo Kim, Heejun Park

**Affiliations:** 1 College of Pharmacy, Duksung Women’s University, Samyangro 144-gil, Dobong-gu, Seoul 01369, Republic of Korea; amyjon@naver.com (J.J.); jinny00@duksung.ac.kr (J.J.); joohee@duksung.ac.kr (J.J.); hyosun1102@duksung.ac.kr (H.C.); songseoul17@duksung.ac.kr (K.S.); eskim19@duksung.ac.kr (E.-S.K.); sanghyup@duksung.ac.kr (S.H.L.); homepage2600@duksung.ac.kr (E.H.); whchung23@duksung.ac.kr (W.-H.C.); armoon@duksung.ac.kr (A.M.); ktkang@duksung.ac.kr (K.-T.K.); 2 College of Pharmacy and Research Institute for Drug Development, Pusan National University, Busandaehak-ro 63beon-gil, Geumjeong-gu, Busan 46241, Republic of Korea

**Keywords:** nose-to-brain drug delivery system, donepezil, Alzheimer’s disease, intranasal

## Abstract

Donepezil (DPZ) is an Alzheimer’s disease (AD) drug that promotes cholinergic neurotransmission and exhibits excellent acetylcholinesterase (AChE) selectivity. The current oral formulations of DPZ demonstrate decreased bioavailability, attributed to limited drug permeability across the blood–brain barrier (BBB). In order to overcome these limitations, various dosage forms aimed at delivering DPZ have been explored. This discussion will focus on the nose-to-brain (N2B) delivery system, which represents the most promising approach for brain drug delivery. Intranasal (IN) drug delivery is a suitable system for directly delivering drugs to the brain, as it bypasses the BBB and avoids the first-pass effect, thereby targeting the central nervous system (CNS). Currently developed formulations include lipid-based, solid particle-based, solution-based, gel-based, and film-based types, and a systematic review of the N2B research related to these formulations has been conducted. According to the in vivo results, the brain drug concentration 15 min after IN administration was more than twice as high those from other routes of administration, and the direct delivery ratio of the N2B system improved to 80.32%. The research findings collectively suggest low toxicity and high therapeutic efficacy for AD. This review examines drug formulations and delivery methods optimized for the N2B delivery of DPZ, focusing on technologies that enhance mucosal residence time and bioavailability while discussing recent advancements in the field.

## 1. Introduction

### 1.1. Donepezil Development History

Donepezil (DPZ) is a reversible acetylcholine esterase (AChE) inhibitor that increases brain acetylcholine (ACh) concentration and promotes cholinergic neurotransmission to improve brain memory, cognition, and learning ability [1,2]. The biological half-life of DPZ is 70 h, and its oral bioavailability is close to 100% [1,3]. In addition, DPZ has the distinct advantages of high AChE selectivity, strong efficacy, and low toxicity. Given these traits, DPZ is now commonly prescribed as a primary drug for mild to moderate Alzheimer’s disease (AD). First-generation cholinesterase inhibitors (ChEI) such as physostigmine and tacrine used before DPZ improved patients’ cognitive function, but had disadvantages such as poor pharmacokinetic profiles (physostigmine) and severe hepatotoxic side effects (tacrine). To develop a drug that could overcome these shortcomings, research on DPZ was initiated in 1983 at Tsukuba Research Laboratories, Eisai Co., Ltd., Japan. The core hypothesis of this research and development was the “cholinergic hypothesis,” which states that abnormal cognitive decline and memory impairment in individuals with AD are due to a lack of cholinergic neural connections in the basal forebrain [4,5,6]. To solve this problem, Eisai researchers attempted to develop several compounds that could inhibit the breakdown of ACh, resulting in indanone derivatives that had a long half-life and a moderate AChE activity inhibition effect. Among these, Eisai selected E2020 or DPZ Hydrochloride (HCl) and began joint development with Pfizer. The two companies obtained approval from the United States Food and Drug Administration (USFDA) for DPZ under the name “Aricept” in 1996, and launched it in Atlanta, USA, in 1997. In 2010, Ranbaxy Lab received approval for a generic version of DPZ from the USFDA, and in 2011, Wockhardt also received provisional marketing approval from the USFDA [1,7]. DPZ is a second-generation ChEI that selectively inhibits AChE, prevents degradation of ACh at synapses in both the central nervous system (CNS) and peripheral nervous system (PNS), and does not cause hepatotoxicity. These characteristics of DPZ suggest that DPZ has overcome the limitations of earlier first-generation ChEIs [6,8].

As mentioned above, DPZ, which plays an important role in the treatment of AD, is also showing potential as a treatment for other neurocognitive disorders, especially vascular dementia. Clinical trial results show that DPZ can improve cognitive function in vascular dementia patients through its cholinergic mechanism. Additionally, DPZ is demonstrating potential in managing neurological disorders that involve cognitive impairment. Examples of such neurological disorders include autism spectrum disorder, Parkinson’s disease, traumatic brain injury, and post-stroke cognitive impairment. But Clinical trials showed that there were neurological disorders for which DPZ was not effective, such as Down syndrome [6,9,10], occipital cortical atrophy [6,11], depression (Table 1) [6,12,13].

### 1.2. Physicochemical Characteristics and Clinical Considerations of Oral DPZ

**Figure 1:**
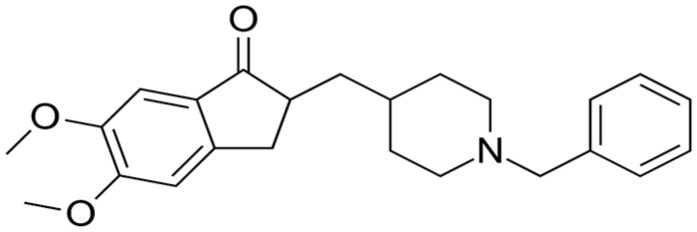
Chemical structure of DPZ.

DPZ HCl has a molecular weight of 425.96 g/mol and logP of 4.27. It contains a tertiary amine group with a pKa of 8.9 and is a white to off-white solid (Figure 1) [14]. The melting point was 224 °C according to thermal analysis [15,16]. It is soluble in chloroform, water (55 g/L at 25 °C), and glacial acetic acid. The USFDA has approved DPZ HCl, and various studies are being conducted to apply the free base form (DPB) of DPZ as an active drug. Unlike DPZ, DPB is insoluble in water (33 g/L) and has a melting point of 85.6 °C as determined by differential scanning calorimetry [17]. Therefore, DPB may be suitable for the development of lipid-based dosage forms, but further studies on the efficacy and safety of DPB are still needed [6].

When managing patients with AD, it is common to start with 5 mg initially and increase to 10 mg after 4 to 6 weeks, and this method reduces adverse drug reactions. The usual maintenance dose prescribed for patients with mild AD is 5 mg per day. For patients with moderate to severe AD who have taken 10 mg of DPZ daily for more than 3 months, a 23 mg sustained-release formulation can be applied.

When comparing the two doses, 23 mg/day and 10 mg/day, in terms of efficacy and safety, the 23 mg/day dose was associated with a higher incidence of adverse events (AEs) compared to the 10 mg/day dose in terms of cognitive function measured by the Severe Impairment Battery (*p* < 0.01). In addition, the 23 mg/day dose had a higher incidence of AEs after administration than the 10 mg/day dose (66.7%), but most AEs were mild to moderate, and serious AEs were rare (<1.0%). As a result, the 23 mg/day dose had a greater cognitive improvement effect than the low dose, but was characterized by an increase in mild to moderate AEs [6,18]. Moreover, in one study, approximately 30% of patients who took the 23 mg dose withdrew from the trial due to intolerable adverse events, while only 18% of patients who took the 10 mg dose withdrew [19]. Therefore, the use of high-dose (23 mg) DPZ may be limited in actual clinical practice due to AEs [6,18].

There remain unresolved challenges in the clinical use of oral DPZ formulations, particularly with regard to side effects. In clinical trials, the most frequently reported adverse events, occurring in at least 2% of patients at a higher rate than placebo, include nausea (11%), diarrhea (10%), insomnia (9%), muscle cramps (6%), fatigue (5%), vomiting (5%), and anorexia (4%). These side effects are more common in patients taking higher doses of DPZ. The increase in gastric acid secretion, associated with cholinergic activity of DPZ, has been linked to gastrointestinal bleeding, with approximately 1% of patients reporting GI hemorrhage. However, these side effects tend to subside within 1–3 weeks of continuous use and are often alleviated by a 6-week titration period. Careful monitoring is recommended when non-steroidal anti-inflammatory drugs are used concurrently due to the potential for adverse effects [6].

Despite the availability of DPZ, its effectiveness in treating AD remains uncertain, largely due to the complex structure of the brain, particularly the presence of the blood–brain barrier (BBB). This barrier prevents most external substances from entering the brain, resulting in low brain delivery efficiency of many drugs, including DPZ, due to poor transport across the BBB [1,20,21,22,23]. Following oral administration of DPZ, the ability of DPZ to exert its pharmacological effects in the central nervous system is critically influenced by the blood–brain barrier (BBB), which regulates its penetration into the brain by limiting passive diffusion and actively modulating drug transport via efflux mechanisms such as P-glycoprotein [24,25,26,27].

### 1.3. Various Strategies for Overcoming the Limitations of DPZ Oral Delivery

To overcome these challenges, research has been actively pursued into new formulations of DPZ and alternative routes of administration. These efforts aim to address the limitations of current oral tablet formulations, such as side effects, and improve the efficiency of drug delivery to the brain [28]. Furthermore, several novel dosage forms and advanced delivery technologies have been developed to enhance the therapeutic efficacy of DPZ, as explained below.

#### 1.3.1. Rapid Disintegration Tablets and Oral Film

Drugs with a fast-dissolving drug delivery system (DDS) can avoid the first-pass effect, have rapid drug effects, and have high bioavailability. Examples of these include orally disintegrating tablets (ODTs) and orally disintegrating films (ODFs) [29].

In the case of ODT, they disintegrate in the mouth without the need for water or chewing. Therefore, they are advantageous in increasing medication compliance in patients who feel uncomfortable taking tablets with water, patients with swallowing problems, and patients who have difficulty swallowing existing oral preparations [30]. In fact, guardians of AD patients tended to prefer ODTs over DP film-coated tablets [31]. Taking advantage of these advantages, USFDA-approved drugs are being released in many countries [32]. Examples of ODT include DPZ ODT Tab, Aripe ODT, and Teva DPZ ODT [33].

However, ODT also has various disadvantages. Insoluble disintegrants added to orally disintegrating tablets can produce an unpleasant taste. In addition, in the case of AD patients, orally disintegrating tablets can cause drug resistance. Also, if the tablet is compressed too strongly during manufacturing, it takes a long time to disintegrate, and if it is compressed too weakly, there is a problem with stability during storage or transportation, so it is necessary to find an appropriate compression force in the middle. Because of these problems, many researchers have started to study ODFs. ODFs have a thin and large surface area, so they disintegrate faster in the mouth than ODTs and are quickly absorbed into the body. In addition, the taste can be easily adjusted compared to ODTs, so it can mask the bitter taste. In addition, ODFs can be preferred over ODT because they maintain the advantages of not needing water when taking them, not needing to be swallowed, and being able to directly deliver the drug without the first-pass effect, which can increase bioavailability. Examples of USFDA-approved DPZ ODF drugs include Neurocept ODF, Neutoin ODF, and Bearcept ODF [33,34]. However, ODF formulations have disadvantages such as difficulty in achieving uniform drug distribution and potential difficulties in controlling the drug release profile [32].

#### 1.3.2. Patches

Transdermal patches offer several notable advantages as DDS. They are noninvasive or minimally invasive medicated adhesive, allowing the drug to diffuse through the epidermis into the systemic circulation, facilitating drug delivery across the skin [35]. This approach bypasses first-pass metabolism, thereby increasing bioavailability and allowing controlled or sustained drug release [36]. Transdermal patches can also improve patient compliance due to their ease of use [34,36]. Ongoing development and clinical trials of DPZ transdermal patches aim to leverage these advantages to provide a more consistent drug delivery option with fewer gastrointestinal side effects [6,37].

For example, Valia et al. proposed two types of patches: drug reservoir-in-adhesive and drug matrix-in-adhesive. In particular, drug matrix-in-adhesive has the characteristic of rapid controlled release of drugs from the reservoir layer to the adhesive layer [38]. Additionally, Kim and Gwak found that the diffusion rate was high in formulations containing various solvents and diffusion-promoting agents, especially isopropyl alcohol [39]. Saluja et al. improved the skin permeability of DPZ gel patches using an iontophoresis system. This method increased the drug delivery rate and maximum blood concentration (C_max_), and showed a longer half-life than intravenous injection [14]. Galipoglu et al. developed a DPZ biopolymer-based transdermal film using sodium alginate and a diffusion enhancer [40]. As the transdermal administration method is likely to be an excellent choice for DPZ HCl administration, many researchers are currently researching and developing DPZ transdermal patches, as in the example above [41].

However, there are several disadvantages to drug administration through transdermal patches. The primary limitation of transdermal DDSs is the barrier properties of the skin, which significantly restrict drug penetration. Only drugs with specific physicochemical properties, such as molecular weights below 1000 daltons and sufficient solubility in both water and oil, are suitable for transdermal delivery. Furthermore, the development of transdermal patches requires careful consideration of drug stability, skin irritation, and sensitization, ensuring consistent drug release and precise dosage. The use of permeation and penetration enhancers is often necessary to overcome the skin barrier, which temporarily alters the stratum corneum’s structure to allow drug absorption [42,43,44,45,46]. However, these enhancers carry the risk of causing skin irritation or allergic reactions, presenting a challenge to achieving safe and effective transdermal drug delivery. These limitations reduce patient compliance with medication [34].

#### 1.3.3. Microneedle

Microneedles, characterized by their micron-scale dimensions (generally less than 1 mm), create microscopic perforations in the epidermis to facilitate drug delivery through the skin [32]. In the context of delivering DPZ HCl, the drug was incorporated into the tips of dissolvable microneedles, which were manufactured using micro molding techniques. DPZ HCl was solubilized in hydroxypropyl methyl cellulose (HPMC) at various concentrations, and the microneedle tips were fabricated by blending DPZ HCl-loaded HPMC with ethanol and water, while the bases were composed of carboxymethyl cellulose and water. This approach aims to enhance skin permeability by circumventing the stratum corneum, a primary barrier to drug absorption [32].

The efficacy of this system was evaluated through insertion tests conducted with porcine skin, utilizing three microneedle arrays, each containing different concentrations of DPZ HCl [32]. Upon insertion, the drug-loaded tips partially dissolved, enabling the diffusion of DPZ HCl into the skin [32]. Pharmacokinetic data demonstrated that a significant amount of DPZ HCl was encapsulated in the microneedle tips, with over 95% of the drug delivered within 5 min and complete dissolution of the tips occurring within 15 min. Additionally, the C_max_ achieved with the microneedle system was four times greater than that observed with oral administration. The authors concluded that the tip-loaded microneedle system represents a promising transdermal delivery method for DPZ HCl, potentially offering improved patient acceptance [6,47].

As such, microneedles have the advantage of being able to quickly reach a high drug concentration because they directly pierce the skin and deliver drugs. However, there are also some limitations. Some swelling around the injection site with the use of microneedles, along with common problems such as skin allergies, redness, and irritation, can occur [48]. The microneedles are so small that the tips can break, and complications may occur if the fragments remain in the skin. In addition, microneedles have limited drug loading capacity and have difficulty delivering large or hydrophilic compounds through the skin. Therefore, it is important to set the appropriate mechanical strength and insertion force to minimize pain and increase drug permeability. However, it may be difficult for patients to initially prick the skin with microneedles, and there is a possibility of infection if the skin pores do not close after application [49,50].

#### 1.3.4. Long-Acting Injection (LAI)

Traditional injection methods, such as intravenous injection, subcutaneous injection, and intramuscular injection, have the advantage of rapidly increasing the blood drug concentration in the body through rapid absorption of the drug while allowing accurate administration of the drug [51,52]. However, in the treatment of AD, it is important to maintain a stable and continuous blood drug concentration, so a method different from the existing injection method is needed. Therefore, the development of a long-acting injection (LAI) of DPZ is highly necessary. If a LAI is developed, it can maintain a stable blood drug concentration, thereby reducing rapid changes in blood drug concentration and related side effects. In addition, it can also relieve the inconvenience of AD patients who often have difficulty chewing and swallowing, having to take oral medication every day [53].

As demonstrated by various developments and clinical examples of long-acting injectables, Poly (D,L-lactide-glycolide) (PLGA) is extensively utilized in DDSs due to its biodegradability and biocompatibility [54]. Examples of PLGA-based DDS include microspheres, nanoparticles, and implants, which can provide sustained drug release ranging from days to months. Clinically, PLGA-based microspheres offer several advantages, such as prolonged drug action, complete biodegradability within the body, excellent biocompatibility, and the potential to enhance the bioavailability of specific drugs, including proteins [32,55,56,57].

LAI technologies face several ongoing challenges. When developing fixed-dose combinations involving multiple drugs, optimization is required to maintain consistent drug ratios over extended periods, considering the distribution coefficients of each drug [58,59]. Liquid formulations may necessitate wide-bore needles or multiple injections due to high viscosity. Additionally, the size and shape of the formulation post-injection can vary depending on factors such as tissue structure and injection technique, affecting drug release rates [60]. Suspension formulations may lead to issues with drug particle stability and variability in particle size distribution [61]. Manufacturing and sterility control remain persistent challenges, requiring advanced processing technologies for micronized particles and high-concentration liquids [62,63]. These technological challenges significantly impact the efficient development and application of LAIs [64].

### 1.4. Nose-to-Brain (N2B) Drug Delivery of DPZ

As previously discussed, numerous delivery systems have been explored, yet they still face limitations in several areas. To address these challenges, nose to brain (N2B) is emerging as a potential solution.

DPZ is usually administered peripherally, in which case the drug is selectively introduced into the CNS by the BBB. The BBB reduces drug permeability into the brain, making it difficult to achieve sufficient drug concentrations. This reduces the efficacy of treating CNS disorders such as AD [65]. Although the BBB has the clear advantage of protecting the brain, it has the disadvantage of not being able to increase drug concentrations in the brain sufficiently to achieve therapeutic effects for neurological disorders [66]. To overcome these limitations, methods such as intranasal (IN) administration that bypass the BBB and enhance CNS-targeted therapeutic effects are being explored [1,32,67,68,69].

#### 1.4.1. N2B Drug Delivery Mechanism and Advantages

IN drug delivery has been a promising method to directly deliver drugs to the brain by bypassing the BBB through the nose. This approach was first proposed by William H. Frey II in 1989 [70]. Since then, much progress has been made in the development of N2B DDS [71].

The nasal cavity is divided into the vestibular, respiratory, and olfactory regions, each of which plays an important role in drug delivery [72]. The vestibular region is rich in mucus and ciliated cells, which can hinder drug delivery through mucociliary clearance [73]. The respiratory region is rich in blood vessels and contains trigeminal sensory neurons, which contribute to drug delivery to the brainstem and cerebrum [73]. The olfactory region plays a role in directly delivering drugs to the brain. Nasally administered drugs first encounter the mucociliary clearance mechanism of the vestibular region, and mucoadhesive systems, surface-engineered nanocarriers, etc., are used to overcome this [74].

**Figure 2:**
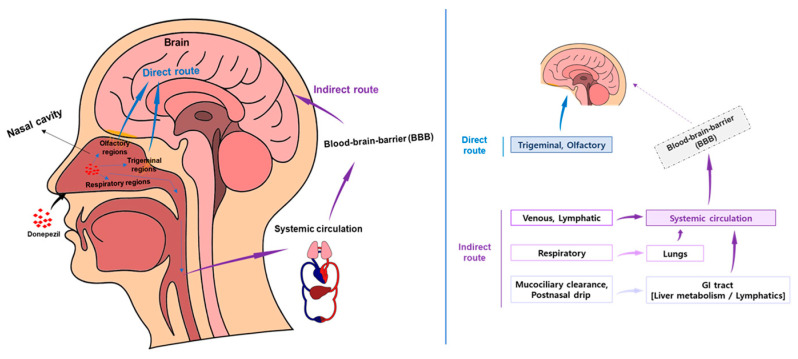
N2B drug delivery pathways of DPZ through olfactory, trigeminal, and systemic routes.

Figure 2 shows that the olfactory and trigeminal pathways are the main mechanisms for delivering drugs from the nasal cavity to the brain, enabling rapid delivery by bypassing the BBB [75,76,77,78,79]. In particular, highly hydrophobic drugs are effective for brain targeting through this route. Efficient nasal drug delivery is hindered by several physiological barriers, such as mucociliary clearance, limited surface area for absorption, and enzymatic activity in the nasal mucosa. These challenges necessitate the use of formulation strategies including mucoadhesive polymers, bioadhesive gels, and nanocarriers designed to prolong residence time and enhance drug penetration. Such approaches are particularly important for optimizing central nervous system targeting via the nose-to-brain route [80].

The IN route can directly deliver drugs to the brain by bypassing the BBB, and there are three main routes. First, the olfactory pathway is where olfactory receptor neurons directly deliver drugs to the brain via the olfactory bulb [77]. There is also the trigeminal pathway, where the trigeminal nerve delivers drugs from the oral cavity, nasal cavity, and eye to various brain regions [77]. Through the systemic route, drugs can be rapidly absorbed into the bloodstream through the blood vessels of the non-mucosal membrane and transported to the brain [81]. The N2B delivery route has advantages over oral administration, intravenous injection, and direct brain injection, such as patient convenience, BBB bypass, and avoidance of first-pass metabolism, and is attracting attention as an effective method for treating neurological diseases. There are several well-demonstrated review papers about physiological pathways facilitating drug transport to the brain following intranasal administration. We suggest referring to those review papers [1,25,76,78,80,82,83,84].

#### 1.4.2. Key Issues in the Development of N2B Drug Delivery Formulation Technology

IN drug delivery is increasingly attracting attention as a method for rapid drug delivery to the brain due to its advantages of bypassing the BBB, rapid onset of drug effects, and avoidance of first-pass metabolism [83,85]. However, there are still several challenges for the clinical application of this method. Major limitations include low drug permeability through the nasal mucosa, rapid mucociliary clearance, enzymatic degradation, short drug retention time, and potential nasal mucosal toxicity [83]. In addition, due to the limitations of the nasal cavity size, it is not suitable for drug delivery that requires large doses or continuous administration [73,86].

Various strategies have been designed to overcome these problems. Representative examples include permeation enhancers, controlled-release systems, and colloidal drug carriers to improve drug absorption and extend nasal retention time [87,88,89]. In addition, mucoadhesive systems, high viscosity formulations, mucoadhesive polymers, and hydrogels have been utilized to reduce mucociliary clearance and improve drug retention [90]. Nevertheless, the effectiveness of nasal drug delivery still varies depending on the physicochemical properties of the drug and the properties of the formulation [91]. For example, although some studies have reported success in direct N2B delivery of certain drugs, a study from the University of Leiden did not find evidence of such delivery for compounds such as estradiol and vitamin B12, indicating the need for further research and understanding of the N2B pathway [91,92]. Given these remaining challenges, current research focuses on optimizing drug formulations and delivery methods to increase the clinical applicability of N2B drug delivery [93,94]. In the following, we address specific drug delivery technologies for N2B delivery of DPZ and highlight recent advances and their clinical implications [1].

## 2. Case Studies of DDS for DPZ Delivery to N2B

Currently, prior research on the N2B DDS for DPZ has investigated lipid-based, solid particle-based, solution-based, and gel- and film-based formulations [95,96]. Table 2 provides a summary of the formulations and research results of the DPZ delivery system designed for targeting the nasal. Lipid-based formulations studied include emulsions, micelles, liposomes, lipid nanoparticles, liquid crystals (cubosomes and hexosomes), while solid particle-based formulations investigated comprise inhalable dry powders and suspensions. In addition to this, formulations such as gels and films have been proposed, and this discussion will address research cases aimed at enhancing brain delivery efficiency.

### 2.1. Lipid-Based Formulations

Lipid-based N2B DDSs are advantageous in drug delivery for central system diseases due to their low toxicity and stability. Also, they prolong the residence time of the drug on the nasal mucosa, avoiding the rapid mucociliary clearance, and enhance the adhesion to the olfactory epithelium. In particular, the types of lipids are more important to a formulation study. Thus, appropriate selection of the lipids can result in high encapsulation efficiency, bioavailability, and an increase in the residence time.

**Figure 3:**
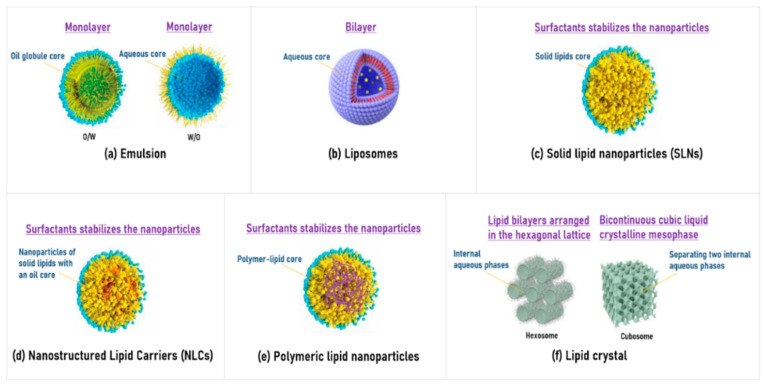
Lipid-based nanocarrier and characteristics: (**a**) oil-in-water (O/W) and water-in-oil (W/O) emulsions; (**b**) liposomes; (**c**) solid lipid nanoparticles (SLNs); (**d**) nanostructured lipid carriers (NLCs); (**e**) polymeric lipid nanoparticles; (**f**) liquid crystals. (**a**,**c**–**e**) are adapted and slightly modified from Ref. [95]; (**b**,**f**) are adapted and slightly modified from Ref. [96]. All images are used under the terms of the Creative Commons CC BY 4.0 license.

Lipid-based formulations include representative systems such as emulsions, liposomes, solid lipid nanoparticles (SLNs), nanostructured lipid carriers (NLCs), polymeric lipid nanoparticles, and liquid crystal nanoparticles. Their structural and compositional characteristics are summarized in the following Figure 3 [93,94,95].

#### 2.1.1. Emulsion

**Figure 4:**
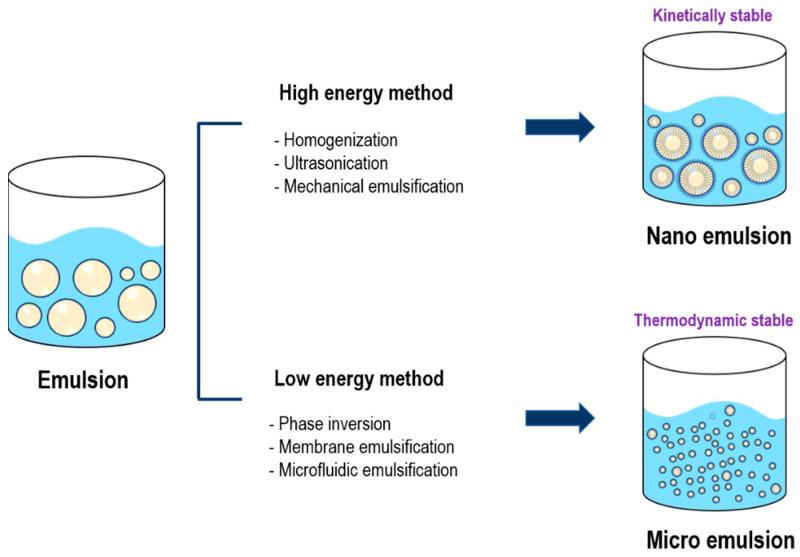
Overview of emulsification techniques used for the preparation of NE and ME.

Microemulsion (ME) and Nanoemulsion (NE), which are widely used for N2B drug delivery, can bypass the BBB through the olfactory and trigeminal nerve pathways [135]. Figure 4 shows the preparation methods and stability characteristics of NE and ME for drug delivery. NEs are typically produced using high-energy emulsification techniques such as high-pressure homogenization, ultrasonication, and mechanical stirring [136,137]. MEs have relatively smaller droplet sizes and are thermodynamically stable systems formed through low-energy emulsification methods, including phase inversion, membrane emulsification, and microfluidic techniques. Emulsions can improve the solubility of hydrophobic drugs and prevent drug degradation by nasal enzymes, protecting against enzymatic degradation, controlling drug release, and increasing bioavailability [138]. NE can also be formulated with mucoadhesives such as chitosan, which can extend the retention time of drugs in the nasal mucosa and improve drug absorption into the brain. NE consists of oil, surfactant, and cosurfactant, and this composition plays an important role in the performance of NE [139,140]. Surfactants, co-surfactants reduce interfacial tension to increase the fluidity of the formulation and stabilize NE. In general, as the concentration of co-surfactant increases, the sphere size of NE decreases, and the drug concentration increases. Zeta potential affects the colloidal stability and mucosal adhesion of the formulation. Positively charged NE adheres better to the negatively charged nasal mucosa, thereby improving drug retention [141,142,143]. Firstly, studies using NE as a formulation have been described below.

Espinoza et al. developed a NE with a combination of bioadhesion and PNE (penetration-enhancing properties of nanoemulsion) of DPZ [69]. The formulations, DPZ-NE and DPZ-PNE, were prepared by a stirring method at 700 rpm, and the size of DPZ-NE was 128.50 ± 1.03 nm. In vitro results showed high encapsulation efficiency (DPZ-NE, 94.32 ± 0.12%, DPZ-PNE, 93.85 ± 0.095%), and in the ex vivo permeation profile, the amount of drug permeated through the nasal mucosa was higher for DPZ-PNE at 532.30 µg compared to DPZ-NE at 199.56 µg. Furthermore, in the ex vivo mucoadhesion study, DPZ-PNE exhibited improved mucoadhesion with a value of 82.43 ± 1.72%, surpassing the 71.31 ± 1.53% of DPZ-NE. Moreover, in vivo studies using a nasal spray pump in pigs showed no erythrocyte and inflammatory cell infiltration and no lamina propria changes. Therefore, the higher mucoadhesion rate and in vivo safety of DPZ-PNE suggest an extended residence time at the absorption site. They concluded that using a polymer for the IN administration of DPZ would effectively address the existing permeation and residence time issues [69].

In the study by Kaur et al., an O/W NE of DPZ was developed, containing 10% Labrasol, 1% cetyl pyridinium chloride (1% in 80% water), and 10% glycerol [97]. The NE was prepared by vortexing, pre-homogenization, and ultrasonication methods, and the average particle size was 65.36 nm. In vitro, the NE showed rapid release, and the cell viability was 76.3%. In vivo studies, NE administered intranasally to rats exhibited a C_max_ of 3.42%/g in the brain and 2.04%/g in the blood, indicating a higher absorption rate in the brain compared to both oral and intravenous routes. This indicates that the IN route is the most effective for targeting the drug [97].

In a study conducted by Handa et al., a NE for co-delivery of DPZ and memantine was developed using low-energy emulsification [98]. The formulation used pine oil as the lipid phase, polysorbate 80 as a surfactant, and diethylene glycol monoethyl ether as a co-surfactant. In vitro, the formulation was spherical in size and had a zeta potential of −7.22 mV. In vivo results in mice showed that after 15 min of IN administration, the brain drug concentrations were approximately 678 ng/mL for DPZ and approximately 249 ng/mL for memantine, which were more than twice as high as those from other routes of administration. In contrast, the plasma concentrations were approximately 3 ng/mL for DPZ and approximately 28 ng/mL for memantine after 15 min, indicating a lower concentration. Thus, this suggests that NE can serve as a promising alternative that overcomes existing administration forms by delivering a greater amount of the drug when administered through IN routes [98].

Secondly, research cases regarding ME formulations have been presented as follows. A ME based on DPZ was developed, and its delivery to the brain via the IN route was studied by Lupe et al. Castor oil, Labrasol, Transcutol-P, and propylene glycol were used in the manufacturing process, and the O/W emulsification method was used [99]. The average droplet size of the final product was 58.9 ± 3.2 nm, and it had a spherical, smooth surface. In vitro tests showed that the total release of DPZ was 74.66% after 24 h, and the release mechanism followed the hyperbolic kinetic model. In ex vivo studies, the highest permeation was observed during the first 4 h, and after 6 h, the residual amount of DPZ per tissue was 812.9 μg/g, corresponding to 32.52% of the initial dose [99].

Another study was conducted by Khunt et al. and used butter oil (BO) and omega-3 fatty acid-rich fish oil (O3FO) as components of DPZ HCl-ME. These were prepared by the physical mixing method [100]. The globule sizes of the prepared DPZ-BO-ME and DPZ-HCl-O3FO-ME were 87.66 ± 5.23 nm and 88.59 ± 8.23 nm, respectively. In vitro studies, DPZ-HCl-BO-ME and DPZ-HCl-O3FO-ME showed high nasal diffusion with the ratios of 71.22 ± 1.21% and 62.16 ± 1.23%, respectively, and in vivo studies, the drug bioavailability in the brain of DPZ-HCl-BO-ME and DPZ-HCl-O3FO-ME increased to 313.59 ± 12.98% and 361.73 ± 15.15%, respectively. Considering the cases mentioned above, it can be inferred that ME and NE as N2B DDS can improve bioavailability [100].

#### 2.1.2. Liposome

Liposomes, spherical nano-sized vesicles derived from cholesterol and phospholipids, have been widely studied in the field of IN drug delivery for systemic and brain-targeted therapy. These vesicles are ideal carriers of therapeutic agents because they exhibit high safety and biocompatibility. When administered intranasally, drugs encapsulated in liposomes primarily enter the systemic circulation via the respiratory tract, but can also be directly transported to the brain via the olfactory pathway. This direct route allows targeted delivery to the CNS by bypassing the BBB. Liposomes offer several advantages, including protection of drugs from nasal degrading enzymes, enhanced drug absorption across the nasal epithelium, and prolonged residence time in the nasal cavity. The large surface area and high solubility of nano-sized particles, which are generally less than 1000 nm, contribute to effective nasal absorption and stability. Research has focused on liposomes because of their similarity to biological membranes composed of cholesterol and phospholipids, and their potential for safe and efficient drug delivery has been highlighted [75,144,145,146,147,148]. The following cases are provided to support this explanation.

Asmari et al. prepared liposomes for DPZ using the thin layer hydration technique, incorporating cholesterol, polyethylene glycol (PEG), and 1,2-distearyl-sn-glycero-3-phosphocholine [101]. The liposomes, with a size of 102 ± 3.3 nm, exhibited high encapsulation efficiency (84.91% ± 3.31%) and a polydispersity index (PDI) of 0.28 ± 0.03. In vivo studies in male Wistar rats demonstrated improved bioavailability and sustained release of DPZ HCl via IN administration, with a longer half-life (6.90 ± 1.14 h) compared to the free drug (5.55 ± 1.04 h), and no significant toxicity [101].

Also, in a study by Amarjitsing Rajput & Shital Butani, liposomes loaded with DPZ HCl were used [105]. The ethanol injection method was used as the preparation method. The liposomes were spherical in shape, 103 ± 6.2 nm in size, 0.108 ± 0.008 in polydispersity, and 93 ± 5.33% incorporation efficiency. In vitro, the optimized in situ gel containing these liposomes showed 80.11 ± 7.77% drug permeation through sheep nasal mucosa, whereas the permeation rate of the in situ gel based on DPZ HCl solution was 13.12 ± 4.84%. In vivo, the formulation showed significantly high brain concentration of 1239.61 ± 123.60 pg/g after IN administration, indicating the potential of IN administration for the treatment of AD, a brain disorder [105].

In the study by Salem et al., hyaluronic acid-coated transfersomes (DPZ HCl-HA-TFS) containing DPZ HCl were selected as a DDS [103]. It was formulated using a thin film hydration method optimized through a 24-factorial design. DPZ HCl-HA-TFS had a vesicle size of 227.5 nm and an entrapment efficiency of 75.83%. The cumulative release was 37.94% after 8 h in vitro, and the nasal mucosa penetration was 547.49 µg/cm^2^ after 24 h. The formulation exhibited adequate stability and nontoxicity. The drug targeting index was 5.08, the drug targeting efficiency was 508.25%, and the direct delivery ratio from N2B was 80.32% [103]. They concluded that liposomes can be considered a promising technology for the N2B delivery of DPZ as a long-term non-invasive carrier.

#### 2.1.3. Solid Lipid Nanoparticles (SLNs)

SLNs were developed as submicron colloidal carriers (50–1000 nm) for the delivery of hydrophilic and lipophilic drugs encapsulated in a biocompatible material core. The core is composed of lipids and stabilized by surfactants present in the outer shell. SLNs offer advantages such as targeted drug delivery, sustained release, and enhanced bioavailability. Additionally, they exhibit superior biodegradability, have no acute or chronic toxicity, and can be produced on a large scale. SLNs can protect encapsulated drugs from biological and/or chemical degradation, and their closure effect, superior application properties, and mucoadhesive characteristics can prolong nasal residence time, thereby improving drug delivery from the nose to the brain. Thus, the following cases presented studies that utilized SLNs for N2B DDSs [104].

Yasir et al. developed SLN by blending two surfactants. SLN of DPZ were prepared using a mixture of glyceryl monostearate as lipid and tween 80 and poloxamer 188 (1:1) as surfactants via the solvent emulsification diffusion method [104]. The optimized formulation had a particle size of 121.0 nm, zeta potential of −24.1 mV, entrapment efficiency of 67.95% and drug loading of 12.15%. The formulation exhibited an in vitro drug release profile of 89.35%. In an in vivo study on male albino Wistar rats, AUC_0-∞_ in the brain was increased by 2.61-fold compared to intravenously administered DPZ-Sol, suggesting significantly higher bioavailability and demonstrating its utility in the treatment of AD (*p* < 0.05) [104].

In this study by Topal et al., SLNs were developed to enhance the delivery of DPZ HCl to the brain [105]. The formulation used tripalmitin as the lipid, Tween 80 as the surfactant, and lecithin as the cosurfactant. SLNs were prepared using homogenization and sonication techniques. The optimized SLNs had a particle size of 87.2 ± 0.11 nm, a PDI of 0.22 ± 0.02, and an encapsulation efficiency of 93.84 ± 0.01%. In vitro release studies showed that the cumulative release of DPZ HCl was approximately 70% after 24 h. The particle size and PDI increased with increasing lipid concentration, but decreased with increasing amounts of surfactant (Tween 80) and cosurfactant (lecithin). The particles were spherical and smooth, and no cellular toxicity was observed [105].

The investigation carried out by Yasir et al. reveals that DPZ-SLNs were formulated using glyceryl behenate as the lipid and a blend of Tween 80 and Poloxamer 188 (1:1) as surfactants via the solvent emulsification diffusion technique [106]. The optimized DPZ-SLNs exhibited a particle size of 96.72 ± 5.89% drug release over 24 h, with stability maintained at 4 ± 2 °C and 25 ± 2 °C/60 ± 5% relative humidity (RH) for six months. In vivo studies in albino Wistar rats showed an AUC_0-∞_ for brain tissue of DPZ-SLNs that was 1.97 times higher compared to DPZ-Solution administered intravenously, with a drug targeting efficiency of 288.75% and a direct transport percentage of 65.37% [106].

However, despite their high targeting efficiency, SLNs are susceptible to drug leakage during storage due to their high moisture content and crystalline lattice integrity. To address this issue, a liquid lipid-containing NLC system has been developed as follows [149].

#### 2.1.4. Nanostructured Lipid Carriers (NLCs)

NLCs are lipid carriers composed of a mixture of solid and liquid lipids, stabilized by surfactants in the outer layer. Introducing liquid lipids improves the flexibility of the solid lipid matrix, leading to the formation of imperfections in the liquid crystal arrangement. This results in increased space for drug encapsulation and improved stability compared to SLNs. NLCs demonstrate higher encapsulation efficiency than SLNs and can capture both hydrophilic and lipophilic drugs. Furthermore, most of the lipids used in the formulation of lipid nanoparticles are physiologically compatible and biodegradable, preventing the accumulation of degradation products in the brain. The size of NLCs allows for close contact with the stratum corneum, promoting adhesion and increasing hydration, making them suitable for N2B systems. The following are cases related to NLCs [107,110].

Firstly, Yasir et al. designed DPZ-loaded NLCs for brain targeting [107]. The lipids were Compritol and Capryol 90, the surfactant was poloxamer 188, and the coating agent was chitosan, which were prepared using homogenization and sonication techniques. The particle size was 192.5 ± 7.3 nm, the entrapment efficiency was 89.85 ± 2.17%, and the PDI was 0.298 ± 0.021. In vitro studies showed spherical particles and an amorphous drug form in the NLC matrix. In vivo results revealed a two-fold increase in the permeability coefficient (10.14 ± 1.73 × 10^−3^ cm h^−1^) compared to DPZ-Solution, a 2.02-fold higher bioavailability after IN administration, and significantly improved brain targeting efficiency (321.21%) and drug transport percentage (74.55%) [107].

Secondly, Ali et al. developed DPZ HCl and Embelin-loaded NLCs [108]. These were prepared using the hot emulsification probe sonication method. The diameter of the NLCs, optimized through Quality by Design (QbD)-based Central Composite Rotatable Design, was 180.2 nm, the PDI was 0.37, and the zeta potential was −12 mV. In vitro, the NLCs showed sustained drug release of 90.72 ± 1.00% for DPZ HCl and 81.30 ± 0.52% for Embelin over 24 h, which was in accordance with the Korsemeyer–Peppas model. In vitro studies showed excellent nasal mucosal penetration with a fluorescence depth of up to 35 μm. Histopathological evaluations showed that the NLCs used in this study were non-toxic and non-irritating [108].

Thirdly, Tekade et al. developed NLCs of DPZ HCl. The formulation consisted of glyceryl monostearate as a solid lipid, Tween 80 and poloxamer 407 as surfactants, and Nigella sativa oil as a liquid lipid [109]. The NLCs were prepared by high-pressure homogenization followed by ultrasonication. The prepared NLCs had a particle size of 107.4 ± 2.64 nm, a dispersion index of 0.25 ± 0.04, and a zeta potential of −41.7 mV. In vivo studies showed that the maximum brain concentration was 4.597 µg/mL after 1 h, and the blood concentration was 2.2583 µg/mL. This indicates the preferential nasal penetration and brain distribution of DPZ HCl [109].

Finally, Shehata et al. prepared NLCs using glyceryl palmitostearate (solid lipid) and oleic acid (liquid lipid) using a high-shear homogenization technique [110]. DPZ/Astaxanthin–NLC containing 10 mg DPZ and 40 mg astaxanthin had a z-average diameter of 149.9 ± 3.21 nm, a dispersion index of 0.224 ± 0.017, and a zeta potential of −33.7 ± 4.71 mV. In vitro, both drugs were released for 24 h and stable for 6 months at 4–8 ± 2 °C. In vivo, these NLCs significantly reduced neuroinflammatory markers in an AD-like mouse model and showed excellent cognitive function improvement, which was better than when DPZ–NLC alone was administered [110].

#### 2.1.5. Polymeric Lipid Nanoparticles

Polymeric lipid hybrid nanoparticles are core–shell structures that consist of a polymeric core surrounded by lipid or lipid–PEG shells, combining the physical stability and biocompatibility of both polymeric nanoparticles and liposomes. Due to recent advancements in nanomedicine, these nanoparticles have become a highly effective and promising nanocarrier for various biomedical applications, including drug delivery and biomedical imaging [150].

Expandable polymeric lipid nanoparticles technology was employed in the study of Bhandari et al. They developed hybrid polymer lipid nanoparticles using chitosan and gelatin as natural polymers in their study [111]. The nanoparticles were prepared by combining these polymers with lecithin. The average particle size of chitosan lecithin was 237.43 nm, and that of gelatin lecithin nanoparticles was 278.86 nm. In vitro release studies showed that chitosan lecithin nanoparticles released DPZ explosively up to 99.99% over 5 days, whereas gelatin lecithin nanoparticles showed sustained release of 33.31% over 30 days under acidic conditions. Both formulations were found to be mucoadhesive and safe on mouse fibroblast cells (L929). Gelatin lecithin nanoparticles showed more potent drug delivery potential than chitosan lecithin nanoparticles [111].

#### 2.1.6. Liquid Crystal (Cubosome and Hexosome)

Liquid crystals are an intermediate phase between the solid and liquid phases. Characterized by the presence of nanocrystals, they exhibit the properties of both phases. Liquid crystals demonstrate anisotropic characteristics and are generally elongated molecules with non-uniform electrical charge distribution. Lipids that form Liquid crystals, such as phospholipids, fatty acids, triglycerides, and cholesterol, are prepared in aqueous solution with appropriate surfactants to produce dispersions such as lamellar, hexagonal, and cubic phases. Among them, cubosomes, which correspond to the cubic phase, have excellent advantages as nanoparticles prepared with low viscosity by homogeneously dispersing a bicontinuous cubic phase with high viscosity. They are colloidally and thermodynamically stable, and their large internal surface area makes them favorable for encapsulating drug molecules [151,152]. Thus, Liquid crystals can serve as DDS for the IN administration of DPZ, with the following cases presented.

In a study by Ruela et al., the use of a lyotropic liquid crystal mesophase to enhance the administration of DPZ was studied [17]. The formulation was prepared by direct mixing of monoolein, oleic acid, and water at room temperature and then equilibrated for at least 7 days. The study showed a phase transition from the water-in-oil (W/O) ME to the reverse hexagonal mesophase, with an increase in viscosity and mucoadhesive properties in the porcine intestinal mucosa. In vitro release studies showed a controlled release of DPZ for up to 24 h, with 61.67 ± 1.11% (pH 1.2), 4.33 ± 0.27% (pH 4.5), and 1.16 ± 0.24% (pH 6.8) released at 90 min, depending on the pH [17].

Patil et al. developed a cubosomal mucoadhesive in situ nasal gel using glycerol monooleate and poloxamer 407 for better delivery of DPZ HCl into the brain via the IN route [66]. The cubosomes were prepared through oil-in-water (O/W) emulsification. The particle size ranged from 137.8 nm to 231.4 nm. The zeta potential was −40 mV. In vitro studies showed that the drug entrapment efficiency ranged from 30.85% to 48.48% with an initial burst release in phosphate buffer, followed by a sustained drug release (53.73% after 6 h). In vivo studies in rats showed that the C_max_ and bioavailability of the cubosomal gel formulation were significantly higher (*p* < 0.001) than that of the plain drug solution, and it was retained in the nasal cavity for a long time [66].

de Souza et al. developed a mucoadhesive lyotropic liquid crystal formulation for IN administration of DPZ using CETETH-10, oleic acid, and water (40:45:15, *w*/*w*). The formulation was prepared by mixing, followed by a water bath (42 ± 2 °C) and stirring (2 min), and was maintained for 24 h at room temperature [115]. It showed a phase transition from ME to lyotropic liquid crystal upon contact with artificial nasal fluid (12–20%). In vitro studies showed that the mucoadhesive strength was 6.41 Ns with 20% ANF. The drug was released at a sustained rate of 25% for 6 h. In vivo studies in Wistar rats showed that the formulation maintained DPZ concentrations in the brain above 1000 ng/g for up to 4 h, with C_max_ reached at 1.5 h [115].

In a study by Dhiman et al., liposomes containing both DPZ and pine oil were formulated using egg lecithin through the thin film hydration technique, followed by sonication [113]. The resulting liposomes had a particle size of 129 nm, PDI of 0.19, zeta potential of −27.5 mV, and were stable at both 4 °C and 25 °C. Morphologically, the oil-loaded liposomes were crystalline cubic in shape, in contrast to the spherical liposomes loaded with drug alone. In vitro, this combination showed higher AChE inhibition. In vivo, the liposomes showed rapid cellular uptake without nasal ciliary toxicity [113].

### 2.2. Solid Particle-Based Formulations

#### 2.2.1. Inhalable Dry Powder

Inhalable dry powder formulations that deliver from the N2B often provide increased stability and do not require preservatives. Upon administration, the powder adheres to the moist nasal mucosa, dissolving slowly and allowing sustained absorption. Bioadhesive excipients or agents that slow ciliary action are often used to improve absorption and reduce clearance. Key factors affecting deposition and absorption include moisture sensitivity, solubility, particle size, and flow characteristics. The fabrication of inhalable drug particles has been achieved through distinct engineering strategies, broadly classified into ‘bottom-up’ methodologies—such as spray drying, spray freeze-drying, and supercritical fluid (SCF) techniques—and ‘top-down’ approaches, most notably particle size reduction via milling [153,154]. Delivery devices typically operate via a pressurized powder atomizer, breath-actuated inhaler, or nasal inhaler. These systems efficiently deliver powders into the nasal cavity, and some use breath-actuated technology to optimize distribution and absorption [155].

In the study by Purushottam Gangane and Pravin Kawtikwar, gellan gum-based nasal mucoadhesive microspheres loaded with DPZ HCl were prepared using pine oil, polysorbate 80, ethanol, PEG, DGME, and TDW [114]. The preparation method was conventional spray drying. The particle size was optimized using a full factorial design, and in vitro studies showed zero-order drug release at pH 6.6. The in vitro mucoadhesion ranged from 80.30% to 94.43%. The ex vivo permeation study using sheep nasal mucosa indicated enhanced drug absorption due to the small microsphere size. The results showed sustained drug release and improved IN drug delivery efficiency [114].

Perkušić et al. developed a dry powder formulation for nose-to-brain delivery of DPZ HCl using chitosan and mannitol microspheres prepared via spray-drying [115]. The ultrasonic nozzle enabled the production of particles predominantly larger than 10 µm, enhancing localized nasal deposition. In vitro studies demonstrated a high drug deposition profile of 65.5% in the olfactory region. The formulation showed favorable properties for swelling, mucoadhesion, and drug release. Results indicated potential for safe and efficient delivery, with further in vivo studies recommended for final proof-of-concept [115].

Jadhav et al. synthesized mucoadhesive microparticles of DPZ HCl using chitosan and carbopol 934 via spray-drying. The microparticles had mean particle sizes of 18.3–21.4 µm for chitosan and 14.7–18.3 µm for Carbopol [118]. In vitro studies showed drug release percentages of 66.57–85.74% for chitosan and 69.54–91.53% for carbopol. In vivo studies with Albino rats demonstrated higher brain drug concentrations for chitosan (110.87 ± 6.87%) and carbopol (129.51 ± 9.82%) compared to the pure drug, indicating enhanced delivery through the IN route [118].

#### 2.2.2. Suspension

Suspensions are colloidal dispersions of nano- to microsized drug particles stabilized by surfactants. It can be applied to enhance the solubility of water-insoluble drugs and is one of the preferred formulations for the IN administration [156]. In this session, we will examine cases of N2B research related to DPZ using nanosuspensions and microsuspensions, and discuss the results [120].

(1) Nanosuspension

Nanosuspension is a colloidal dispersion of drug particles in a liquid medium, with a particle size of less than 1 μm. Nanosuspension improves the water solubility and lipid permeability of drugs, facilitating brain delivery, especially for drugs that have difficulty crossing the BBB. Nanoparticulate suspensions can effectively target drugs to specific sites in the body due to their very small size, allowing them to penetrate capillaries. When nanoparticles are manufactured using biodegradable and biocompatible materials such as PLGA, sustained drug release at the targeted site becomes possible. Relevant studies related to this topic are described below [120].

Bhavna et al. developed DPZ-loaded PLGA nanoparticles using the solvent emulsification diffusion–evaporation technique [120]. The nanoparticles were formulated with a 50:50 PLGA block copolymer (35–40 kDa) at various drug-to-polymer ratios (1:1, 1:2, 1:5, and 1:10 *w*/*w*). The drug-loaded nanoparticles exhibited a size of 89.67 ± 6.43 nm and displayed a spherical shape with smooth morphology. In vitro release studies indicated a biphasic pattern, featuring an initial burst followed by sustained release. In vivo biodistribution showed significantly higher brain radioactivity per gram for the nanoparticulate formulation compared to the drug solution (*p* < 0.05). Coated nanosuspensions suggest a significant improvement in the absorption rate of DPZ in the brain, potentially leading to enhanced treatment outcomes for AD [120].

Bhavna et al. developed a DPZ-loaded chitosan nanosuspension using an ionic-crosslinking method [85]. The nanosuspension had an average particle size of 150–200 nm and a PDI of 0.341. In vitro studies revealed a drug loading capacity of 40–48% and a cumulative release percentage ranging from 56.17 ± 5.26% to 96.74 ± 0.65% over 300 min. In vivo, IN administration in Sprague-Dawley rats showed higher AUC values in plasma (684.83 ± 15.43 ng h/mL) and brain (352.75 ± 10.68 ng h/mL) compared to the control group, with no observed toxicity or significant changes in body weight and hematological parameters [85].

Also, several researchers performed research on NLPs using chitosan, as follows.

Garg et al. (2024) designed DPZ HCl-loaded chitosan nanoparticles using the ionotropic gelation method [121]. The formulation comprised DPZ HCl, chitosan (medium molecular weight), sodium tripolyphosphate, glacial acetic acid. The nanoparticles had a size of 177.8 nm, a zeta potential of +16.6 mV, and a drug payload of 22.2 mg DPZ HCl/100 mg chitosan. In vitro studies showed sustained drug release for 24 h, with 90.82% release and 2.29 times higher flux compared to DPZ HCl solution. In vivo studies in Wistar rats demonstrated 2.6 times greater drug delivery to the brain after 6 h compared to the control, with no significant changes in particle size, drug content, or zeta potential over 3 months [121].

Lupe et al. developed IN gels of DPZ using different formulations DPZ-Chitosan-Gel (DPZ-CGel) and DPZ-Pluronic F-127-Gel (DPZ-PGel1 and DPZ-PGel2) [122]. The gels were prepared with varying methods, including the use of glycol monoethyl ether (Transcutol^®^ P) and N-methylpyrrolidone. In vitro results showed that DPZ-PGel1 had the highest release rate (>98%) and flux values, with a maximum release amount of 2249 µg, compared to 81.8% release and 1615 µg for DPZ-CGel. DPZ-PGel1 also exhibited better permeation and retention in porcine nasal mucosa, with a significant improvement in bioavailability. The formulations demonstrated suitable physicochemical properties, and cytotoxicity was not apparent [122].

Yogesh Garg and Amit Bhatia developed chitosan nanoparticles loaded with DPZ HCl using the ionic gelation method [124]. The nanoparticles had an average particle size of 180.2 nm, a zeta potential of +16.6 mV, and a PDI of 0.282. In vitro studies showed over 90% drug release and more than 70% drug permeation within 24 h, adhering to the Korsmeyer–Peppas model for sustained release. In vivo, IN administration of the nanoparticles led to a threefold increase in drug delivery to the rat brain compared to the drug solution, with confocal microscopy confirming greater localization in the brain [124].

Garg et al. developed chitosan nanoparticles for nose-to-brain delivery of DPZ HCl using a QbD approach [123]. The nanoparticles had an average size of 180.2 nm, a PDI of 0.282, and a zeta potential of +16.6 mV. In vitro drug release showed more than 90% drug release with sustained release following the Korsemeyer–Peppas model. Ex vivo permeation studies revealed over 70% drug permeation within 24 h. In Wistar rats, IN administration of these nanoparticles resulted in approximately 2.6 times more drug in the brain compared to IN solution and about 10 times more than oral solution after 6 h. Therefore, they concluded that chitosan nanoparticles can be used as drug carriers to facilitate brain delivery [123].

In this study, Handa et al. developed mannose-coated PLGA nanoparticles for delivering DPZ and memantine [119]. The nanoparticles were prepared using a simple emulsification method, with a final size of 179.4 nm and a zeta potential of −33.1 mV. The concentration of drugs in the brain was ~573 ng/mL for memantine and 207 ng/mL for DPZ via the IN route, showing a 3-fold increase compared to uncoated nanoparticles. The IN route also demonstrated superior efficacy in neurobehavioral assessments, gene expression analyses, and biochemical estimations. These findings suggest that the IN route could be a promising alternative for delivering therapeutic agents in neurological treatments [119].

(2) Microsuspension

In the N2B research on DPZ using microsuspension, microspheres were primarily utilized. Microspheres are water-insoluble carriers that swell as they absorb water into their matrix, leading to the formation of a gel. The materials primarily used in this context include starch, dextran, albumin, and hyaluronic acid, and these formulations can be utilized in a variety of routes of administration. Particularly in IN DDSs, they act directly on the mucosa, allowing penetration through and between the tight junctions of epithelial cells. This technology has the potential to improve nasal mucosal permeability and extend residence time, as evidenced by the results presented in the following research studies [157].

Ambadkar et al. developed mucoadhesive microspheres for IN delivery using HPMC K4M and K15M combined with Gellan Gum through a W/O emulsification cross-linking technique [117]. The microspheres had an average particle size ranging from 157.65 μm to 373.87 μm, with entrapment efficiencies between 34.5% and 53%. In vitro studies showed production yields of 44.26% to 55.93%, drug loading of 3.45% to 5.23%, swelling properties of 0.82% to 0.91%, and drug release of approximately 98% within 7 h. The HPMC-based microspheres demonstrated suitable mucoadhesion without damaging the nasal mucosa [117].

Gangane et al. developed IN mucoadhesive microspheres of DPZ HCl using gellan gum as the polymer [116]. The microspheres were prepared via a W/O emulsification cross-linking technique, involving gentle heating and constant agitation (40 °C, 1700 rpm). The resulting microspheres exhibited a particle size ranging from 14.3 to 18.3 µm. In vitro studies showed a drug loading of 49.21% to 74.60%, entrapment efficiency of 34.5% to 53.6%, and a swelling percentage of 82% to 91%. The in vitro drug release and ex vivo permeation profiles were 76.92% to 98.26% and 60.76% to 95.73%, respectively, over 300 min [116].

In conclusion, the suspension is the most extensively studied formulation for the N2B system of DPZ, suggesting that it is a viable alternative to existing administration forms and methods.

### 2.3. Solution-Based Formulation

Briefly, a solution is a formulation made using a solvent that can dissolve the Active Pharmaceutical Ingredient (API) [158]. The IN route of administration is typically solution, as water-soluble formulations aerosolize well, and the manufacturing process is relatively simple. However, stability issues such as precipitation of the API may occur during product storage, and preservatives may cause irritation to the nasal mucosa. Currently, suspensions are being studied more than solutions to improve drug solubility and enhance bioavailability [159,160].

Lee et al. evaluated the relative contribution of the direct pathway in brain transport for 17 model drugs with different physicochemical properties [125]. The formulation involved dissolving each drug in a solution containing 10 mg/mL of the compound in a mixture of PEG 400, ethanol, water for injection, or dimethyl sulfoxide, with 250 mL of the solution administered to 250 g rats. In vivo results showed that five of the model drugs were significantly delivered to the brain via the direct pathway. The study found no statistically significant correlation between physicochemical properties and direct pathway transport, suggesting the potential role of carrier-mediated transport in nasal drug delivery [125].

### 2.4. Gel

Recent advances in nasal drug delivery have underscored the significance of mucoadhesive and nanogel systems in enhancing formulation performance [161,162,163]. These systems prolong mucosal residence time and facilitate drug permeation across the nasal epithelium, thereby improving the efficiency of N2B delivery for donepezil.

Hydrogels exhibit mucoadhesive properties through a cross-linked network using polymers such as chitosan and poloxamers. The mucosal adhesion effect of the gel improves the bioavailability of the drug by prolonging its residence time in the nasal mucosa. However, the high viscosity of the gel significantly hinders aerosolization, reducing drug delivery efficiency. Thus, gel formulations require an accurate drug delivery device to overcome this challenge. Despite these characteristics, this formulation has attracted interest in recent research due to its potential to enhance the nasal mucosal residence time of DPZ [127,164]. Especially, nanogels represent a class of nanosized hydrogel systems characterized by a highly crosslinked internal structure, which may be derived from either monomers or preformed polymers [165]. Their average particle size generally falls within the 20–200 nm range, allowing for favorable properties in biomedical applications [166,167]. These nanogels are increasingly recognized as versatile drug delivery vehicles due to their robust colloidal stability, high drug-loading efficiency, and stimulus-responsive behavior. Their structural adaptability and enhanced permeability confer distinct advantages over traditional nanomaterials, particularly in promoting drug transport to the central nervous system [168].

In the study by Dr. Shital Butani, an ion-sensitive in situ gel was formulated using NLCs containing DPZ [126]. The NLCs were prepared via a melt emulsification-probe sonication method, incorporating glyceryl distearate, Capmul MCM, Acrysol K150, Poloxamer 188, Tween 80, gellan gum, and xanthan gum. The resultant particles had a size of <200 nm, with a PDI of <0.300, a zeta potential of −35 mV, and exhibited a viscosity range of 2–11 cp. In vivo studies in scopolamine-induced amnesia rats demonstrated that the in situ gel achieved a higher C_max_ in the brain and lower C_max_ in plasma compared to the marketed formulation, with a time of maximum concentration (T_max_) of 1 h, and a significantly lower drug concentration in the liver than when administered orally. These findings suggest enhanced safety and efficacy when administered via the nasal route [126].

Al Harthi et al. prepared liposomal DPZ HCl dispersed in thiolated chitosan hydrogel for IN delivery [127]. They used chitosan, 2-iminothiolane HCl, 2-mercaptoethanol, and HCl, employing radiation-induced crosslinking for preparation. The liposomes had a mean size of 438.7 ± 28.3 nm and an entrapment efficiency of 62.5% ± 0.6. In vitro studies showed a half-life of 3.5 h, with better stability at 4 °C compared to 20 °C. In vivo results in rabbits demonstrated a 46% increase in C_max_ and a 39% increase in AUC with IN delivery, and more than a 2-fold increase in brain drug content compared to oral tablets [127].

Fugen Gu’s study developed a DPZ HCl thermosensitive in situ gel using Poloxamer 407 and Poloxamer 188 [129]. The gel was prepared via the cold method, with hydroxypropyl-β-cyclodextrin as a permeation enhancer and ethylparaben as a preservative. The gelation temperature was 32.5 °C with a gelation time of 40 s, and drug release reached 90% within 45 min. In vivo studies showed that the nasal gel had a C_max_ of 4113.41 ng/mL at 10 min, with an AUC of 2718.25 ng h/mL, significantly higher than the oral solution. The relative bioavailability was 385.58%, and brain targeting efficiency was 151.2%. Consequently, nasal gels offer enhanced targeting efficiency in comparison to oral formulations [129].

In the study by Adnet et al., liposomes were prepared using phosphatidylcholine and cholesterol in an 8:1 ratio with chloroform/methanol (4:1) via the thin film hydration method [131]. The liposomes, extruded to a pore diameter of 100 nm, were incorporated into a thermosensitive gel made from Poloxamer 407 and Poloxamer 188. In vitro, the API inhibited butyrylcholinesterase with an inhibitory activity (IC50) of 573 ± 40 nM but had minimal AChE activity (IC50 of 14,520 ± 40 nM). The gel showed a sol–gel temperature of 32–35 °C, an osmolarity of 280 ± 20 mOsm, and a pH of 6 ± 3. The mucoadhesive strength increased with HPMC concentration, reaching 1.0935 dyn/cm^2^. This indicates that the increased viscosity of the polymer can extend the adhesive properties of the gel [131].

In another study, Gangopadhyay et al. developed a DPZ HCl-loaded ethosomal IN gel using phospholipon 90 G and ethanol via the ethanol injection method [128]. The ethosomes had a vesicle size of 110.06 ± 1.910 nm and an entrapment efficiency of 70.02 ± 0.353%. The gel, containing Poloxamer 407 (18%), Poloxamer 188 (6%), and Carbopol 934 (0.1–0.5%), exhibited a gelation temperature of 31.7 ± 0.033 °C, a mucoadhesive strength of 3332 ± 4.314, and nearly 100% drug permeation both in vitro and ex vivo through sheep nasal mucosa after 24 h [128].

Perkušić et al. developed a chitosan-based, DPZ-loaded thermogelling formulation for nose-to-brain delivery [130]. The formulation utilized chitosan (0.5% *v*/*v*), acetic acid, DPZ HCl, and β-glycerophosphate in a solution mixed at 4 °C. The optimized formulation exhibited instant gelation at 34 °C, with an olfactory deposition rate of 71.8% and a prolonged drug release (t1/2 of 90 min). In vitro studies revealed a 20-fold increase in mucoadhesion and a 1.5-fold enhancement in apparent permeability compared to a DPZ solution. Additionally, in vivo testing revealed an acceptable irritability profile in a slug mucosal assay, suggesting the feasibility of safe nasal delivery [130].

### 2.5. Film

The film formulation was proposed to improve the low adherence of the existing solution formulation, sneezing, and inaccurate dosing due to drug leakage. It uses a plasticizer and permeation enhancer to determine the plasticity and flexibility of the film structure, which improves the stability of the drug and the accuracy of dosing due to its robust nature. The outcomes of the researchers who examined the formulation are summarized below [134].

The study by Papakyriakopoulou et al. developed a polymeric nasal film for DPZ HCl using Hydroxypropyl-methyl-cellulose E50 as the film-forming polymer, PEG 400 as a plasticizer, and Methyl-β-Cyclodextrin (Me-β-CD) as a permeation enhancer [132]. The films were prepared via continuous magnetic stirring at 600 rpm and then cooled to 15 °C for polymer hydration. In vitro, the films demonstrated uniform DPZ HCl content (90.0 ± 1.6–99.8 ± 4.9%) and thickness (19.6 ± 1.9–170.8 ± 11.5 µm), with less than 3% residual humidity and favorable swelling and mucoadhesive properties. In vivo studies are ongoing to compare the optimized nasal film’s pharmacokinetics with oral DPZ HCl administration [132].

Kaikousidis et al. developed a nasal film formulation of DPZ HCl using an aqueous film-forming mixture composed of 1.5% HPMC E50, 1.7% PEG 400 (plasticizer), and 0.8% Me-β-CD (permeation enhancer) [133]. The preparation involved dispersing HPMC in hot water (>80 °C), stirring, and cooling (15 °C) for polymer hydration. The film’s in vitro pharmacokinetic parameters included lag time of 4.06 min, absorption duration of 11.3 min, and mean transit time of 56 min. In vivo studies using eight-week-old C57BL/6J mice demonstrated the model’s efficacy in describing drug absorption to both the bloodstream and brain, with parameters reflecting direct nose-to-brain and systemic distribution [133].

In the study by Papakyriakopoulou et al., DPZ HCl was formulated into a nasal film using an HPMC-Me-β-CD-PEG 400-based polymeric system [134]. The film contained HPMC E50, PEG 400 as a plasticizer, and Me-β-CD as a permeation enhancer. It was prepared by dispersing HPMC in hot water (>80 °C), followed by stirring and cooling to 15 °C. In vivo results showed that the nasal film delivered DPZ HCl with C_max_ values 5.7 times higher in the CNS and 3.9 times higher in the bloodstream compared to oral administration, with AUC values of 43.3 and 31.8, respectively. Drug targeting efficiency was 212% and nose-to-brain Direct Transport Percentage was 53%. This suggests that a considerable portion of the IN dose reaches the brain directly through the olfactory or trigeminal nerve pathways [134].

## 3. Expert Opinion on the Commercialization of DZP Formulation for N2B

Although many studies have been conducted for N2B delivery of DPZ, to date, these strategies have remained predominantly within the realm of experimental studies and academic publications. The commercial development considering scalability and high development costs, and regulatory approval of an intranasal formulation of DPZ for N2B drug delivery faces several significant challenges. Clinically, variability in nasal mucosa physiology, potential mucociliary clearance, and limited human data on direct N2B transport may complicate the demonstration of consistent efficacy and safety across patient populations. In particular, the potential safety risks associated with the prolonged use of nanoparticles and various excipients, such as mucoadhesive polymers, warrant thorough investigation. From a regulatory perspective, the absence of established FDA guidelines specific to N2B delivery routes necessitates a case-by-case justification of the formulation’s design, pharmacokinetics, and therapeutic equivalence to existing oral formulations. Approval barriers may also arise due to the need for robust in vivo and clinical evidence confirming enhanced CNS targeting and reduced peripheral side effects. To address these hurdles, sustained and synergistic collaboration across fundamental and applied sciences—including molecular biology, materials chemistry, biomedical engineering, and translational medicine—will be indispensable. Addressing the multifaceted needs of diverse patient cohorts and disease pathologies demands a holistic, cross-disciplinary approach. In more detail, a multi-pronged strategy should be employed, including (i) understanding of drug biodistribution within the brain through considering disease-specific target sites within distinct brain regions and a precise characterization of the pathological mechanisms and molecular targets, (ii) the implementation of standardized methodologies for assessing human pharmacokinetic parameters and/or distribution to ensure reliable and reproducible data interpretation (e.g., the use of advanced imaging and biomarker-based methodologies to track CNS drug deposition), (iii) the development of bio-responsive or mucoadhesive delivery systems to prolong nasal residence time and facilitate direct olfactory transport, (iv) early engagement with regulatory authorities via pre-IND (Investigational New Drug) meetings to align on study design and evidentiary expectations, and (v) the design of specialized intranasal delivery devices suitable for specific drug delivery systems and formulations with a capability of targeting specific anatomical regions within the nasal epithelium. Additionally, conducting comparative efficacy trials against standard oral formulations may strengthen the clinical and regulatory justification for the intranasal route.

## 4. Conclusions

The N2B system of DPZ represents a promising strategy that enhances administration compared to oral tablets and injections while enabling direct delivery by bypassing the BBB. However, issues related to short drug residence time, low drug permeability due to the rapid clearance of the nasal mucosa, and in vivo toxicity need to be addressed. To improve this, various formulations have been developed, with the most research conducted on suspensions to date. Additionally, gel and film formulations related to the N2B system for DPZ have been recently studied, and due to the challenges in formulation, further research is needed to enhance targeting. To summarize the key findings, most in vivo studies have shown higher brain C_max_ and AUC compared to plasma due to improved permeability and mucoadhesion, further enhancing the efficiency of drug delivery to the brain. Furthermore, research on lipid-based formulations resembling biological membranes, as well as solid-based formulations and other formulations such as gels and films designed to enhance stability, indicates cellular non-toxicity and suggests potential safety in vivo. The studies presented earlier have demonstrated improvements in bioavailability, biocompatibility, effective targeting, and sustained release, thus overcoming existing limitations. In the future, it is crucial to further advance research on DDSs for the N2B delivery of DPZ based on these findings. Despite the promising outcomes observed in preclinical studies, several challenges remain to be addressed, including the optimization of dosing strategies, ensuring manufacturing reproducibility, and achieving compatibility with nasal delivery devices. Future research should prioritize resolving these issues to facilitate clinical translation, with particular attention to large-scale production, regulatory approval, and patient adherence. Given the current lack of curative treatments for AD, the successful development of a clinically applicable nose-to-brain formulation of donepezil could represent a major shift in the therapeutic landscape for this condition.

## Figures and Tables

**Table 1:** Emerging clinical indications of donepezil donepezil with proposed mechanisms and reported efficacy.

Indication	Clinical Research Findings	Mechanism/Characteristics	Significant Efficacy	Ref
Alzheimer’s Disease (AD)	Well-established as a major treatment	Acetylcholinesterase inhibition	Yes	[6,13]
Vascular Dementia	Improves cognitive and functional outcomes	Enhances cholinergic neurotransmission similar to AD	Yes	[6,13]
Autism Spectrum Disorder	Under research	Potential cholinergic enhancement	Under Study	[6,13]
Down Syndrome	No significant efficacy observed in clinical trials	Potential cholinergic enhancement	No	[6,9,10,13]
Parkinson’s Disease	Under research	Potential cholinergic enhancement	Under Study	[6,13]
Traumatic Brain Injury	Under research	Potential cholinergic enhancement	Under Study	[6,13]
Post-Stroke Cognitive Impairment	Under research	Potential cholinergic enhancement	Under Study	[6,13]
Multiple Sclerosis-Related Cognitive Impairment	Significant improvements in behavior, cognitive function, and daily activities compared to placebo	Cholinergic enhancement	Yes	[6,13]
Lewy Body Dementia	Significant improvements in behavior, cognitive function, and daily activities compared to placebo	Cholinergic enhancement	Yes	[6,13]
Cortical Atrophy	No significant efficacy observed in clinical trials	Potential cholinergic enhancement	No	[6,11,13]
Depression and Cognitive Impairment	No significant efficacy observed in clinical trials	Potential cholinergic enhancement	No	[6,12,13]
Disease Progression Inhibition	Potential to modify disease (neuroprotective effects)	Inhibition of oxygen-glucose deprivation, Aβ-induced cell death, and glutamate-induced cell death	Under Study	[6,13]

**Table 2:** Overview of formulations and outcomes of DPZ nasal delivery systems.

Drug Delivery System	Features	Formulation	Preparation Method	Result/Outcome		Ref
				In Vitro	In Vivo	
**Lipid-based formulation**						
Emulsion	O/W Nanoemulsion (NE)	- Capryol 90 - Labrasol - Transcutol-P - Water - Pluronic F-127	- Stirring	- Size: DPZ-NE (128.50 nm) - PDI: DPZ-NE (0.12) - pH: DPZ-NE (5.82), DPZ-PNE (6.14) - Transparent, DPZ-NE (monophasic), DPZ-PNE (homogeneous, thermostable) - No signs of precipitated drug, - Entrapment efficiency: DPZ-NE (94.32%), DPZ-PNE (93.85%) - Viscosity: DPZ-NE (10.69 mPa·s) - DPZ-NE (Newtonian behavior), DPZ-PNE (shear thinning behavior) - Permeation profile (porcine nasal mucosa): DPZ-PNE (532.30 µg), DPZ-NE (199.56 µg) - Mucoadhesion study: DPZ-PNE (82.43%), DPZ-NE (71.31%)	- Pigs, IN route, dose: 300 µL - No infiltration of inflammatory cells	[69]
		- Labrasol - Cetyl pyridinium chloride - Glycerol	- Homogenization - Ultrasonication	- Size: 65.36 nm - PDI: 0.084 - Zeta potential: −10.7 mV - Transmittance (100%): 650 nm - Fast release in PBS (pH 7.4), ACSF, simulated nasal fluid - Decreased cell viability: placebo > aqueous DPZ (85%) > NE (76.3%)	- Sprague Dawley rats, IN route, dose: 0.09 mg - Drug distribution: brain > blood - Brain Distribution (1.5 h): NE IN (3.42%/g) > aqueous IN (2.34%/g) > i.v (1.88%/g) > oral (0.58%/g) - Blood Distribution (30 min): i.v (2.97%/g) > IN (2.04%/g) > oral (1.55%/g) - Brain C_max_: NE IN (3.42%/g) > i.v (1.88%/g) > Oral (0.58%/g) - T_max_: NE IN (1.5 h) > i.v (3 h) > Oral (3 h) - Brain retention: NE IN (up to 24 h) > oral (no uptake) - AUC (48.55% h/g), AUMC (619.17% h^2^/g), MRT (12.75 h), kel’ (0.078 h^−1^), CL (0.082%/g h^−1)^, DTE (360.59%), DTP (72.23%)	[97]
		- Pine Oil - Polysorbate 80 - Diethylene Glycol Monoethyl Ether	- Low-energy emulsification	- Size: 16 nm - Spherical - Zeta potential: −7.22 mV	- Swiss albino mice, dose: 1 mg/kg - IN-NE concentration (15 min): Brain (678 ng/mL) > Plasma (3 ng/mL)	[98]
		- Castor oil - Labrasol - Transcutol-P - Propylene glycol	- O/W emulsification	- Size: 58.9 nm - Spherical, smooth, regular surface - PDI: 0.19 - pH: 6.38 - Viscosity: 44.69 mPa·s - Newtonian flow - High stability at temperature variations - Release (24 h): 74.66%, hyperbola kinetic model - Permeation profile (Porcine nasal mucosa): highest permeation during the first 4 h, 80% (2000 μg) - Nasal mucosa retention (6 h): 35.52% (812.9 μg/g)		[99]
	O/W Microemulsion (ME)	- PEG 600, Capmul^®^ MCM EP, Captex^®^ 90, Capryol 90 - Labrasol, Maisine^®^ - PaceolTM - Amyloid β (1–42) - Tween 80, 20, 60 - Transcutol-P^®^ - Butter oil - Omega-3 fish oil	- Water titration	- Size: 52.43–163.10 nm - PDI: 0.26–0.487 - Nasal Diffusion: DPZ HCl-BO-ME (71.22%) > DPZ HCl-O3FO-ME (62.16%) > DPZ HCl-ME (59.69%) > DPZ HCl solution (55.01%)	- Sprague Dawley rats, dose: 0.5 mg/kg - Bioavailability: DPZ HCl-O3FO-ME-IN (361.73%) > DPZ HCl-BO-ME-IN (313.59%) > DPZ HCl-ME-i.v (168.62%) > DPZ HCl solution-IN (8.96%)	[100]
Liposomes	Liposomes	- Carboxymethyl cellulose - 1,2-distearyl-sn-glycero-3-phosphocholine - Cholesterol, PEG - Chloroform - Sodium dihydrogen phosphate	- Thin layer hydration	- Size: 102 nm - spherical shape, single unilamellar vesicle - PDI: 0.28 - Zeta potential: −28.31 mV - Entrapment efficiency: 84.91% - Stability (3 months): stable at 4°, 25 °C	- Male Wistar rats, IN route, dose: 1 mg/kg body weight - Higher AUC_0–t_, AUC_0–∞,_ C_max_ - Sustained release - Higher bioavailability in plasma and brain - Free from toxicity - Half-life: loposome (6.90 h) > free drug (5.55 h)	[101]
	Liposome–based in situ gel	- Hydrogenated soy phosphatidyl cholin - Cholesterol - Ethanol - Ammonium sulfate	- Ethanol injection	- Size: 103 nm - Spherical - PDI: 0.108 - Entrapment efficiency: 93% - Nasal mucosa permeation: DPZ HCl-loaded liposomes (80.11%) > DPZ HCl solution-based in situ gel (13.12%)	- Sprague Dawley rats, IN route, dose: 1 mg/kg - Biodistribution: 1239.61 pg/g, High brain distribution - Brain AUC, T_max_: 1239.61 pg/g, 0.5 h - DTE: 314.29%	[102]
Transfersomes		- Hyaluronic acid	- Thin film hydration	- Size: 227.5 nm - Entrapment efficiency: 75.83% - Release (8 h): 37.94% - Adequate stability - Nontoxic and tolerable for IN delivery - Permeation profile (nasal mucosa, 24 h): 547.49 µg/cm^2^	- Drug targeting index: 5.08 - DTE: 508.25% - Direct N2B DTE: 80.32%	[103]
Solid lipid nanoparticles (SLNs)		- Glyceryl monostearate - Tween 80, poloxamer 188 (1:1)	- Solvent emulsification diffusion	- Size: 121.0 nm - Zeta potential: −24.1 mV - Release: 89.35% - Entrapment efficiency: 67.95% - Drug loading: 12.15% - DSC melting peak: 59.58 C, 54.53 C - Amorphous form - Release: Initial burst, followed by slow release	- Male albino Wistar, IN route, dose: 0.09 mg - AUC_0–∞_: nanoparticle > control group (2.61-fold higher) - Significant (*p* < 0.05) enhancement in bioavailability of DPZ in the brain - Localization (kidney, spleen, liver): nanoparticles > control	[104]
		- Tween 80 - Lecithin	- Homogenization - Sonication	- Size: 87.2 nm - Spherical, smooth - PDI: 0.22 - Zeta potential: −17.0 mV - Encapsulation efficiency: 93.84% - Release (24 h): 70% - Toxicity: no toxic effect on cells - Particle size and PDI increased with increasing lipid concentration but decreased with increasing amounts of surfactant and co-surfactant		[105]
		- Glyceryl behenate - Tween 80, poloxamer 188 (1:1)	- Solvent emulsification diffusion	- Release (24 h): 96.72% - Stability (6 months): no significant change at 4, 25 °C/60% RH (*p* > 0.05), significant particle size increase at 40 °C/75% RH (*p* < 0.001)	- Albino wistar rats, dose: 0.09 mg - AUC_0-∞_: DPZ-SLN >DPZ-Solution (1.92-fold higher) - DTE: 288.75% - DTP: 65.37%	[106]
Nanostructured lipid carriers (NLCs),		- Mixture of Compritol and Capryol 90 - Poloxamer 188 - Chitosan	- Homogenization - Sonication	- Size: 192.5 nm - Spherical shape (TEM. DSC) - PDI: 0.298 - Zeta potential: 38.9 mV - Entrapment efficiency: 89.85%	- Albino Wistar rats, dose: 0.18 mg - Bioavailability (IN): DPZ-chitosan-NLCs > DPZ-Solution - Bioavailability (IN, IV): DPZ-Chitosan-NLCs (IN) > DPZ-Chitosan-NLCs (IV) - Brain Targeting (DTE/DTP): DPZ-chitosan-NLCs (321.21%/74.55%) > DPZ-Solution (158.52%/36.92%)	[107]
		- DPZ HCl and Embelin-loaded	- Hot emulsification probe sonication	- Size: 180.2 nm - Zeta potential: −12 mV - PDI: 0.37 - Release: 90.72% - Embelin: 81.30 - Permeation (goat nasal mucosa): NLCs > suspension - HET CAM score: 0.68 - Cellular uptake study: high cellular uptake of NLC via N2A cells		[108]
		- Glyceryl monostearate - Tween 80 - Poloxamer 407 - Nigella sativa oil	- High-pressure homogenization - Ultrasonication	- Size: 107.4 nm - PDI: 0.25 - Zeta potential: −41.7 mV - Entrapment efficiency: 70.20% - Drug content: 89.05%	- C_max_: brain 4.597 µg/mL > blood 2.258 µg/mL - T_max_: 1 h	[109]
		- Glyceryl palmitostearate - Oleic acid - Astaxanthin - Poloxamer 188 - Polysorbate 80	- High-shear homogenization	- Size: 149.9 nm - Spherical - PDI: 0.224 - Zeta potential: −33.7 mV - Entrapment efficiency: 93.85% - Release (24 h): sustained - Stability (6 months): 4–8 °C		[110]
Polymeric Lipid Nanoparticles		- Soy lecithin - Methanol - Chloroform - Glutaraldehyde - Chitosan - Glacial acetic acid - Ethanol - Gelatin - Acetone	- Homogenization - Desolvation	- Size: CLN (237.43 nm, GLN (278.86 nm) - Drug loading: CLN (10.24%), GLN (8.77%) - Release: CLN (burst release of up to 99.99% drug for 5 days), GLN (sustained release of 33.31% drug for 30 days under acidic conditions) - Cell viability studies; safe toward mouse fibroblast cells (L929) - Mucoadhesive		[111]
Liquid crystal	Cubosomes	- GMO - Poloxamer 407 - Glucomannan - Gellan gum	- O/W emulsification	- Size: 137.8–231.4 nm - PDI: 0.38–0.48 - Zeta potential: −40 mV - pH 6.4 - Entrapment efficiency: 30.85–48.48% - Viscosity: 180 cps - Release (pH 6.6): Initial burst, followed by slow release (24.52% at 2 h, 53.73% at 6 h) - Irregular polyangular (Nearly spherical) - Degree of gelation: Immediate gelation remains for a few hours (less stiff gel) - Mucoadhesive strength: 138.6 g - Gel strength: 34 s - Drug content: 86.07–92.40%	- Male Sprague Dawley rats, IN route, dose: 1 mg/kg - C_max_: OCG 24.01 µg/mL > OCD 14.34 µg/mL > solution 3.96 µg/mL - Bioavailability: OCG, OCD > solution (*p* value < 0.001) - Extended residence time in the nasal cavity - Increased permeation	[66]
	Lyotropic liquid crystal mesophases	- CETETH-10 - Oleic acid - Water	- Mixing - Stirring	- Mucoadhesive Strength (Work of adhesion/Peak of adhesion): M1 (5.67 Ns, 0.93 N), M2 (6.41 Ns, 0.79 N) - Hexagonal phases (12% and 20% nasal fluid): no significant difference in adhesion (*p* > 0.05) - Drug release (6 h): 25%	- Wistar rats, dose: 25 mg.kg^−1^ - Phase transition (12–20% ANF): isotropic to anisotropic - Brain concentration (4 h): sustained above 1000 ng/g	[112]
	Lyotropic liquid crystalline mesophases	- Monoolein/oleic acid/water - Oleic acid - GMO	- Direct mixing - Dissolving	- Swelling study: W/O ME phase to reverse hexagonal mesophase transition - Viscosity increase in situ - Mucoadhesive - Release (24 h): controlled - Dissolution efficiency (90 min): pH 1.2 (61.67%), pH 4.5 (4.33%), pH 6.8 (1.16%)		[17]
	Nano-liquidcrystal	- Pine oil	- Film hydration - Sonication	- Size: 129 nm - PDI: 0.19 - Zeta potential: −27.5 mV - Stability: stable at 4°, 25 °C - Crystalline cubic shape - AChE inhibition activity: higher in combination than others		[113]
**Solid particle-based formulation**						
Microspheres		- Gellan gum	- Conventional spray drying	- Mucoadhesive strength increased as the gellan gum amount increased. - Mucoadhesion range: 80.30–94.43% - Release: zero-order release, likely due to low gellan gum viscosity - Permeation profile (Sheep nasal mucosa): enhanced in smaller microspheres		[114]
		- Chitosan - Mannitol	- Spray drying	- Size: Dv10 (6.7–11.6 μm), Dv50 (17.1–35.7 μm), Dv90 (34.1–72.7 μm) - Hausner ratio: 1.15–1.30 - Spray cone angle range: 22.5–28.3° - Olfactory deposition: 13.9–65.5% - Turbinate deposition: 19.4–47.6% - Drug release (5 h): 100%		[115]
		- Gellan gum	- W/O emulsification cross-linking - Gentle heating - Constant agitation	- Size: 14.3–18.3 µm - Production yield: 40.26–55.93% - Drug loading: 49.21–74.60% - Entrapment efficiency: 34.5–53.6% - Swelling: 82–91% - Mucoadhesion: 45.6–79.6% - Release: 76.92–98.26% - Permeation profile (goat nasal mucosa): 60.76–95.73%		[116]
		- Gellan Gum - N-Octanol - Span 80	- W/O emulsification cross-linking	- Size: 157.65–373.87 μm - Entrapment efficiency: 34.5–53% - Production yield: 44.26–55.93% - Drug loading: 3.45–5.23% - As the stirring rate increased, the particle size decreased - Swelling property: 0.82–0.91% - Release (7 h): 98%		[117]
Microparticles		- Chitosan - Carbopol 934	- Spray drying	- Size: chitosan (18.3–21.4 µm), carbopol (14.7–18.3 µm) - Spherical or ellipsoid - Yield: chitosan (43.96–68.12%), carbopol 56.46–63.23%) - Drug content: chitosan 89.87–96.05%), carbopol (91.5–95.07%) - Drug release: chitosan (66.57–85.74%), carbopol (69.54–91.53%)	- In-bred Albino rats, IN route, dose: 20 µL PBS solution (concentration 50 mg/mL) - Brain distribution: carbopol 934 (129.51%) > Chitosan (110.87%) > Pure DPZ HCl	[118]
Nanoparticles		- India:PLGA (50:50) - Memantine - Polyvinyl alcohol or Tween 80 - span 80 - D-mannose	- Simple emulsification	- Size: 179.4 nm - PDI: 0.22 - Zeta potential: −33.1 mV - SEM: 200 nm - Release: burst release initially, followed by extended release	- Brain distribution (15 min): coated IN (573.61 ng/mL) > uncoated IN (247.77 ng/mL) > coated peroral (168.08 ng/mL) > uncoated peroral (138.27 ng/mL) - Brain AUC, T_max_: 3470.34 ng.h/mL, 0.25 h - Plasma AUC, T_max_: 2526.01 ng.h/mL, 2 h	[119]
Nanosuspension		- PLGA 50:50 block copolymer (35–40 kDa) -Dichloromethane	- Solvent emulsification, diffusion, evaporation	- Size: 89.67 nm - Spherical, smooth (TEM, SEM) - Release: Initial burst, followed by slow release	- Brain distribution: nanoparticles > drug solution	[120]
		- Chitosan - Sodium tripolyphosphate - Glacial acetic acid	- Ionotropic gelation	- Size: 177.8 nm - Drug payload: DPZ 22.2 mg/chitosan 100 mg - Spherical - PDI: 0.593 - Zeta potential: +16.6 mV - Process yield: 91.96% - Release (24 h): sustained, 90.82% - Adhesive force: 9.26 g - Stability (3 months): no significant changes in particle size, drug content, or zeta potential	- Wistar rats, IN route, dose: 40 µL - Brain distribution: nanoparticles > control - High blood clearance	[121]
		- Chitosan - Polysorbate-80 - Acetic acid - Tripolyphosphate	- Ionic cross-linking	- Size: 150–200 nm - PDI: 0.341 - Particle stability (PBS): stable short-term, potential aggregation/size increase long-term - Drug loading capacity: 40–48% - Release (300 min): 56.17–96.74%	- Male Sprague Dawley rats, IN route, dose: 0.5 mg/mL, 1 mg/mL, 1.5 mg/mL (groups I–III) - Plasma, brain AUC: nanosuspension (684.83 ng h/mL, 352.75 ng h/mL) > suspension (440.20 ng h/mL, 95.216 ng h/mL) - Systemic AUC, C_max_, T_max_: Nanosuspension > control	[85]
Suspension	Gel-based suspension	- Chitosan - Transcutol^®^ P - N-Methylpyrrolidone - Water - Pluronic F-127	- O/W emulsification	- pH: DPZ-CGEL (5.9), DPZ-PGel1 (6.2), DPZ-PGel2 (6.3) - Viscosity: at 25 °C, DPZ-CGel > DPZ-PGel1 > DPZ-PGel2; at 35 °C, DPZ-PGel > DPZ-CGel - Swelling (first-order model): DPZ-PGel1 (k = 0.15 min^−1^), DPZ-PGel2 (0.12 min^−1^) - Stability (30 days): no significant changes in its appearance (25, 40 °C) - Release: DPZ-PGel1 (98%) > DPZ-CGel (81.8%) - Maximum release amount: DPZ-PGel1 (2249 µg) > DPZ-PGel2 (1913 µg) > DPZ-Cgel (1615 µg) - DPZ-Pgel1 showed higher values of flux, Kp, partition coefficient vehicle/tissue, diffusion coefficient, and Css - Gelation temperature: 32–33 °C - Nasal mucosa retention, Permeation profile (porcine nasal mucosa): DPZ-PGel1 > DPZ-CGel, DPZ-PGel2		[122]
	Polymeric Nanoparticles	- Chitosan		- Size: 180.2 nm - Spherical (confocal laser) - PDI: 0.282 - Zeta potential: +16.6 mV - Drug release: 90% - Permeation profile (24 h): 70%	- Wistar rats, IN route - Brain distribution (6 h): IN > oral, nanoparticles > solution	[123]
	Nanoparticle	- Chitosan	- Ionic gelation	- Size: 177.8 nm - Zeta potential: +16.6 mV - Drug payload: 22.2 mg/100 mg of chitosan - Process yield: 91.96% - Mucoadhesive strength: 9.26 g - Release (24 h): 90% - Ex vivo release: 70%	- Rats, IN route - 3-fold higher drug delivery to the brain	[124]
**Solution-based formulation**						
Solution		- PEG 400 - Ethanol - Water - Dimethyl sulfoxide	- Dissolving		- Male Sprague Dawley rats, IN route, dose: 4–16 mg/kg - Transporter expression: rOAT3, rOCT2 detected in olfactory epithelium - Brain delivery: direct pathway	[125]
**Gel-based formulation**						
Gels	Ion-sensitive in situ nano gel	- Glyceryl distearate - Capmul MCM - AcrysolK150 - Poloxamer 188 - Tween 80 - Gellan gum - Xanthan gum	- Melt emulsification-probe sonication	- Size: <200 nm - PDI: <0.300 - Zeta potential: −35 mV - Effect of liquid lipid: higher concentration reduced particle size - Expansion coefficient: <3% - Viscosity: 2–11 cp	- Male Sprague Dawley rats, IN route, dose: 1 mg/kg - T_max_: 1 h - Brain C_max_: gel > marketed, - Plasma C_max_: marketed, > gel - Plasma, brain residence time: gel > marketed - Brain drug concentration: IN > oral (2-fold higher)	[126]
	Hydrogel	- Chitosan solution - 2-iminothiolane HCl - 2-mercaptoethanol - HCl - NaCl	- Radiation-Induced crosslinking	- Size: 438.7 nm - Entrapment efficiency: 62.5% - Half-life: 3.5 h - Stability: 4 > 20 °C - Inverse correlation between cross-linking density and swelling degree - Swelling degree: chitosan (21%) hydrogels > thiolated chitosan (19.8%) - Viscosity: chitosan hydrogels > thiolated chitosan hydrogels - Disulfide bonds enhance mucoadhesiveness of thiolated chitosan - TCH no significant histological changes	- New Zealand white rabbits, IN route, dose: 5 mg - C_max_: nasal (12 ng/mL) > oral (8.2 ng/mL) - Brain drug content: IN gel > oral tablets (2-fold higher)	[127]
	Ethosomal nano gel	- Phospholipon - Ethanol - Poloxamer 407, Poloxamer 188 - Carbopol 934	- Ethanol injection	- Size: 110.06 - Entrapment efficiency: 70.02% - Gelation temperature: 31.7 °C - Mucoadhesive strength: 3332 - In vitro drug release - Permeation profile (sheep nasal mucosa): 100%		[128]
	Thermosensitive in situ gel	- Poloxamer 407 - Poloxamer 188 - hydroxypropyl-β-cyclodextrin - Ethylparaben	- Agitation, stirring	- Gelation temperature: 32.5 °C - Gelation time: 40 s - Release (45 min): 90%, sustained, zero-order model	- Sprague Dawley male rats, dose: 10 mg kg^–1^ - Rapid absorption - Bioavailability: 385.58% - Brain DTE: 151.2% - Plasma C_max_, T_max_: gel (4113.41 ng mL^–1^,10 min) > solution (269.35 ng mL^–1^, 55 min) - AUC_0-t_: gel (2718.25 ng h mL^–1^) >solution (735.68 ng h mL^–1^)	[129]
	Thermoresponsive in situ gel	- Chitosan - Acetic acid - β-glycerophosphate solution	- Dropwise, mixing	- Gelation temperature: 34 °C - Olfactory deposition: 71.8% - Release: prolonged drug release (t1/2 about 90 min) - Mucoadhesive behavior - Permeation profile (Calu-3 cells): reversible permeation - Mucoadhesion, Permeation: gel > solution	- Arion lusitanicus slugs - Safety: acceptable slug mucosal irritability profile	[130]
	Thermosensitive gel	- Poloxamer 407, 188 - Phosphatidylcholine/cholesterol (8:1) - Chloroform/methanol (4:1)	- Thin film hydration	- Solubility: higher aqueous solubility - Gelation temperature: 32–35 °C - Osmolarity: 280 mOsm - pH 6 - Viscosity: Shear-thinning behavior with increasing shear rate		[131]
**Film-based formulation**						
Film	Polymeric nasal film	- HPMC E50 nasal films - PEG 400 - Methyl-β-Cyclodextrin	- Continuous magnetic stirring - Cooled for Polymer hydration	- Drug content: 90.0–99.8% - Tthickness: 19.6–170.8 µm - Stability (6 months): stable (airtight), low residual moisture (< 3%) - Enhanced mucoadhesion and nasal permeability		[132]
	Nasal film				- Eight-week-old C57BL/6J mice, IN route, dose: 4 mg/kg - Brain distribution: 0.021 - Blood distribution: 0.005 - T_lag_: 4.06 min, T_k0_: 11.3 min, Mtt: 56 min, k_tr_: 0.171 1/min, P1: 0.543, P2: 0.3, K_a_: 0.173 1/min, K_20_: 0.046 1/min	[133]
	HPMC- Methyl-β-Cyclodextrin-PEG 400 nasal film	- HPMC E50 - PEG 400 - Methyl-β-Cyclodextrin	- Dispersion - Stirring - Cooled for polymer hydration		- Eight-week-old C57BL/6J mice, IN route, dose: 4 mg/kg - C_max_: IN (1.46 μg/mL) > oral (0.37 μg/mL) - AUC_0~t_ (min μg)/mL): IN (43.3) > oral (31.8) - T_max_: IN (15 min) > oral (30 min) - Brain distribution: Film > control	[134]

Abbreviations: O/W, oil in water; DPZ-PNE, penetration-enhancing nanoemulsion (containing Pluronic F-127); DPZ-NE, DPZ-Nanoemulsion; IN, Intranasal; NE IN, Nanoemulsion Intranasal; PDI, polydispersity index; PBS, phosphate-buffered saline; ACSF, artificial cerebrospinal fluid; i.v, intravenous; C_max_, maximum concentration; T_max_, time of maximum concentration; AUC, area under curve; AUMC, area under the first moment curve; MRT, mean residence time; kel’, elimination rate constant; CL, clearance; DTE, drug targeting efficiency; DTP, DPZ HCl-BO-ME, DPZ Hydrochloride-Butter oil-Microemulsion; DPZ HCl-O3FO-ME, DPZ Hydrochloride-Omega-3 fatty acid-rich fish oil-Microemulsion; DPZ HCl-ME, DPZ-HCl-Microemulsion; DPZ HCl, DPZ-Hydrochloride; AUC0–t, area under the plasma concentration-time curve; AUC0–∞, area under the plasma concentration–time curve from 0 h to infinity; DSC, differential scanning calorimetry; DPZ-chitosan-NLCs, DPZ-chitosan-Nanostructured lipid carriers; RH, relative humidity; TEM, transmission electron microscopy; CLN, chitosan lecithin nanoparticles; GLN, gelatin lecithin nanoparticles; GMO, glyceryl monooleate; ME, Microemulsionl; Dv10, Dv50, Dv90, particle diameters at 10%, 50%, and 90% of the cumulative volume distribution; PLGA, Poly (D,L-lactide-glycolide); OCD, optimized cubosomal dispersion; OCG, cubosomal mucoadhesive in situ gel; ANF, artificial nasal fluid; M1, microemulsion optically clear liquid systems + 12% of the ANF; M2, microemulsion optically clear liquid systems + 20% of the ANF; SEM, scanning electron microscope; DPZ-CGEL, DPZ-Chitosan-gel; DPZ-PGEL, DPZ-Pluronic F-127-gel; DPZ-PGel1, DPZ-Pluronic F-127-gel + Transcutol^®^ P (20%); DPZ-PGel2, DPZ-Pluronic F-127-gel + N-Methylpyrrolidone (5%); W/O, water in oil; Kp, permeability coefficient; Css, theoretical plasma concentration at the steady-state in humans; PEG, polyethylene glycol; rOAT3, organic anion transporter; rOCT2, organic cation transporter; TCH, thiolated chitosan hydrogel; T_lag_, lag time; T_k0_, absorption duration; Mtt, mean transit time; k_tr_, transfer constant; P1, fraction of dose absorbed in blood; P2, fraction of dose absorbed in the brain; K_a_, brain absorption constant; K_20_, blood elimination constant.
